# Effects of *CCR5* 59029G/A polymorphism on the risk to diabetic nephropathy

**DOI:** 10.18632/oncotarget.22148

**Published:** 2017-10-30

**Authors:** Mingfeng Cao, Zhenhua Tian, Lin Zhang, Ruiting Liu, Qingbo Guan, Jinjiao Jiang

**Affiliations:** ^1^ Department of Endocrinology, Shandong Provincial Hospital Affilated to Shandong University, Jinan 250100, Shandong, China

**Keywords:** *CCR5*, chemokine, diabetic nephropathy, polymorphism, meta-analysis

## Abstract

**Background:**

Diabetic nephropathy (DN) causes high mortality in patients with diabetes mellitus and imposes heavy burden on individuals and society. In previous studies, various researches have investigated the association of DN with *CCR5* 59029G/A polymorphism, but relevant findings were controversial. Therefore, we performed this meta-analysis to obtain a conclusion on this issue.

**Results:**

*CCR5* 59029G/A polymorphism showed significant risk-increasing effects on DN in all analyses under AA vs. GG, AA+GA vs. GG, AA vs. GG+GA, A vs. G and GA vs. GG model contrasts. Besides, a similar result was also obtained in Asian and type 2 diabetes mellitus groups under these five contrasts after subgroup analyses.

**Methods:**

The relevant publications were searched from the electronic databases and other sources. The association intensity between *CCR5* 59029G/A polymorphism and DN susceptibility was measured using pooled odds ratios (ORs) with corresponding 95% confidence intervals (95% CIs). Inter-study heterogeneity was inspected with Q test, and sensitivity analysis was conducted to verify the stability of the final outcomes by removing one study each time in turn. Begg’s funnel plot and Egger’s test were utilized to examine publication bias among selected studies.

**Conclusion:**

*CCR5* 59029G/A polymorphism is significantly related to enhanced susceptibility to DN, especially in Asian populations and people with type 2 diabetes mellitus.

## INTRODUCTION

Diabetic nephropathy (DN), a common microvascular complication of diabetes, is a major cause of chronic kidney diseases, and results in high mortality in diabetic patients [1, 2]. As a medical condition greatly jeopardizing peoples’ health, DN is a leading cause of end-stage renal disease (ESRD), accounting for approximately half of patients receiving dialysis treatment [3–5]. DN can cause reduced filtration, albuminuria, and ultimately renal failure [6], seriously threatening patients’ lives. During early stage, it can be detected through some changes in renal metabolism and structure [7]. The pathogenesis of DN is complex, and involves multiple pathways, including advanced glycation end products, oxidative stress, inflammatory cytokines and profibrotic factors [8]. There are some methods to detect early changes in diabetic disease, but effective therapeutic measures have not been developed yet [9]. Therefore, it is urgent to ascertain the pathophysiology mechanism of DN for effectively controlling over this disease [10].

Chemokines, inducible proinflammatory cytokines, are regarded as important determinants of early inflammatory responses [11]. The cysteine-cysteine (CC) motif chemokine receptor 5 (CCR5) is involved in immune system where T cells are attracted to specific tissues and organ targets [12], and is constitutively expressed on some immune cells, like T lymphocytes and macrophages [13]. Up-regulated by proinflammatory cytokines, CCR5 acts as a receptor for multiple chemokines, such as chemokine ligand 5 (CCL5), macrophage inflammatory protein-1a (MIP-1a), macrophage inflammatory protein-1b (MIP-1b), and monocyte chemoattractant protein-2 (MCP-2) [14–16]. The gene *CCR5* is polymorphic and located within the gene cluster on chromosome 3p21.3-p24 [17]. The polymorphisms in this gene may affect the activity of the protein, thus contributing to multiple diseases, including HIV-1 infection [18], cancers [19], tuberculosis [20], atopic asthma [21] and DN [22].

Among the polymorphisms of *CCR5* gene, 59029G/A is frequently-reported to be relevant to the susceptibility to DN, but no consensus has been achieved on this issue yet. To systematically explore the effects of *CCR5* 59029G/A polymorphism on susceptibility to DN, we carried out this meta-analysis by pooling the previous findings.

## RESULTS

### Study characteristics

**Figure 1:**
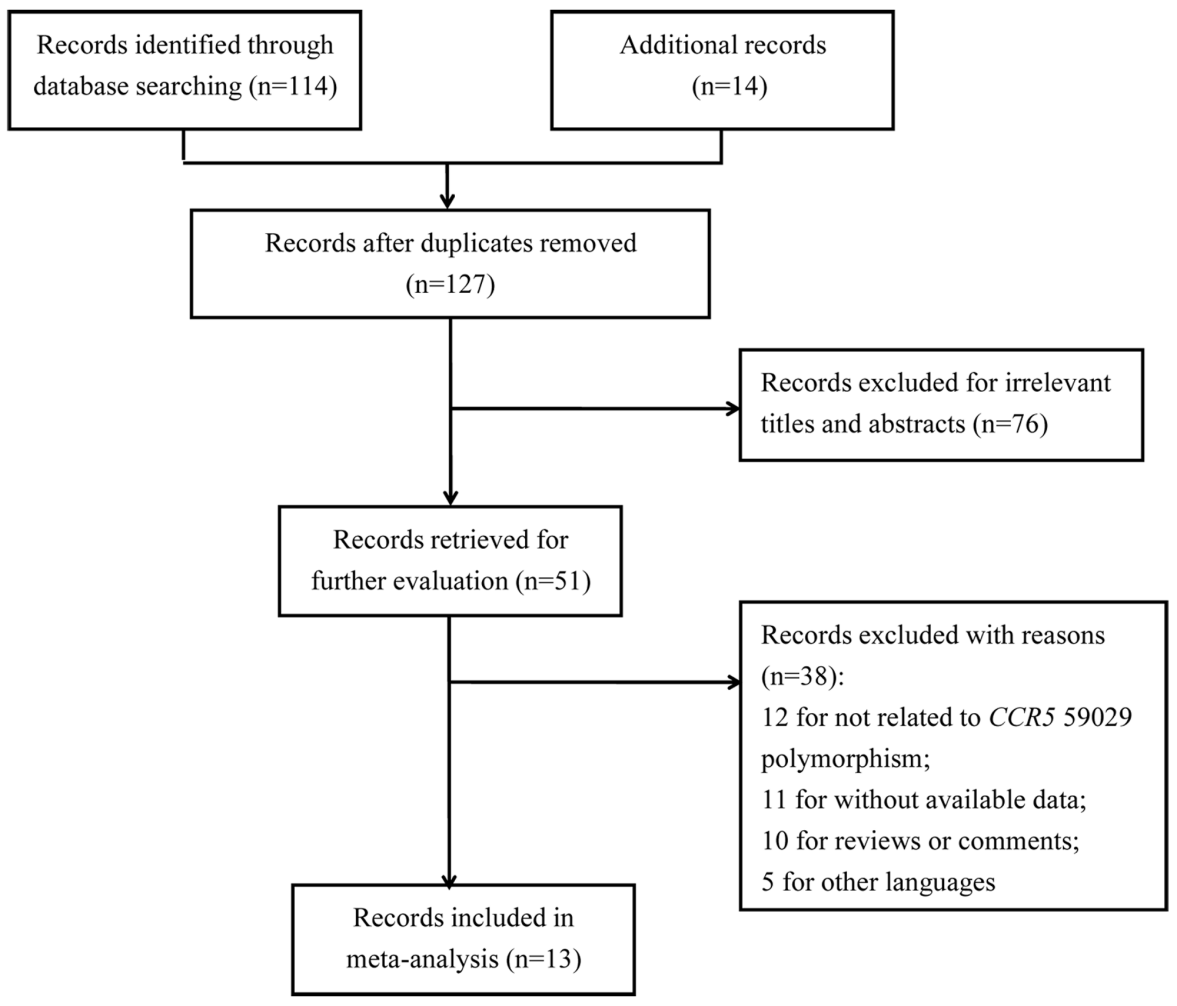
Flow diagram for the process of literature selecting

Initially, a total of 128 publications were retrieved through searching databases and other sources. In literature screening, 115 reports were excluded for duplicate (1), irrelevant title and abstracts (76), and other reasons (38). Among the 38 literature excluded by other reasons, 12 were for not related to *CCR5* 59029 polymorphism, 11 reports were without available data, 10 were reviews or comments, and the resting 5 article were published in other languages (Figure 1). Hence, 13 eligible articles were ultimately incorporated into this meta-analysis [22–34]. Among them, 3 were in Caucasian populations while the others were in Asian populations. Additionally, the quality assessment of these studies based on NOS showed that only the article of Pettigrew [28] had the relative poor quality with 4 scores and the quality the other included studies was good. Table 1 summarized the essential information of all included studies.

**Table 1:** Principal information of studies included in the meta-analysis

Type	First author	Year	Country	Ethnicity	Diagnosis criteria of diabetes	Genotyping method	Case/Control										HWE	NOS
							GG		GA		AA		G		A			
Type 2 Diabetes	Ahluwalia	2009	India	Asian	WHO criteria	PCR-RFLP	40	90	75	80	125	85	155	260	325	250	0.925	8
	Ahluwalia	2009	India	Asian	WHO criteria	PCR-RFLP	20	35	28	30	48	27	68	100	124	84	0.001	8
	Buraczynska	2012	Poland	Caucasian	ADA criteria	PCR-RFLP	15	35	230	100	196	61	260	170	622	222	0.588	6
	Mokubo	2006	Japan	Asian	ADA criteria	PCR-RFLP	12	35	34	50	24	35	58	120	82	120	0.068	7
	Nakajima	2002	Japan	Asian	-	PCR-RFLP	18	67	73	126	41	76	109	260	155	278	0.308	6
	Nakajima	2003	Japan	Asian	EC criteria	PCR-RFLP	41	85	142	173	78	97	224	343	298	367	0.648	6
	Prasad	2007	India	Asian	WHO criteria	PCR-RFLP	27	47	94	111	75	67	148	205	244	245	0.935	5
	Yadav	2014	India	Asian	WHO criteria	PCR-RFLP	78	106	79	80	45	28	235	292	169	136	0.044	6
	Cheng	2010	China	Asian	-	PCR-RFLP	17	25	50	44	27	17	84	94	104	78	0.765	6
	Li	2005	China	Asian	WHO criteria	PCR-RFLP	23	33	42	7	45	13	88	73	132	33	0.827	5
	Wang	2007	China	Asian	WHO criteria	PCR-RFLP	12	19	9	3	18	8	33	41	45	19	0.853	5
	Zhao	2006	China	Asian	WHO criteria	PCR-RFLP	19	28	58	24	43	6	96	80	144	36	0.800	6
Type 1 Diabetes	Mlynarski	2005	Poland	Caucasian	-	PCR-RFLP	104	56	274	152	118	90	482	264	510	332	0.562	8
	Pettigrew	2010	Ireland	Caucasian	-	TaqMan	56	94	129	216	78	127	241	404	285	470	0.904	4

Notes: WHO: World Health Organization; ADA: American Diabetes Association; EC: Expert Committee; PCR-RFLP, polymerase chain reaction-restriction fragment length polymorphism; TaqMan, TaqManSNP; HWE, Hardy-Weinberg equilibrium; NOS: Newcastle-Ottawa Scale.

### Quantitative data synthesis

**Figure 2:**
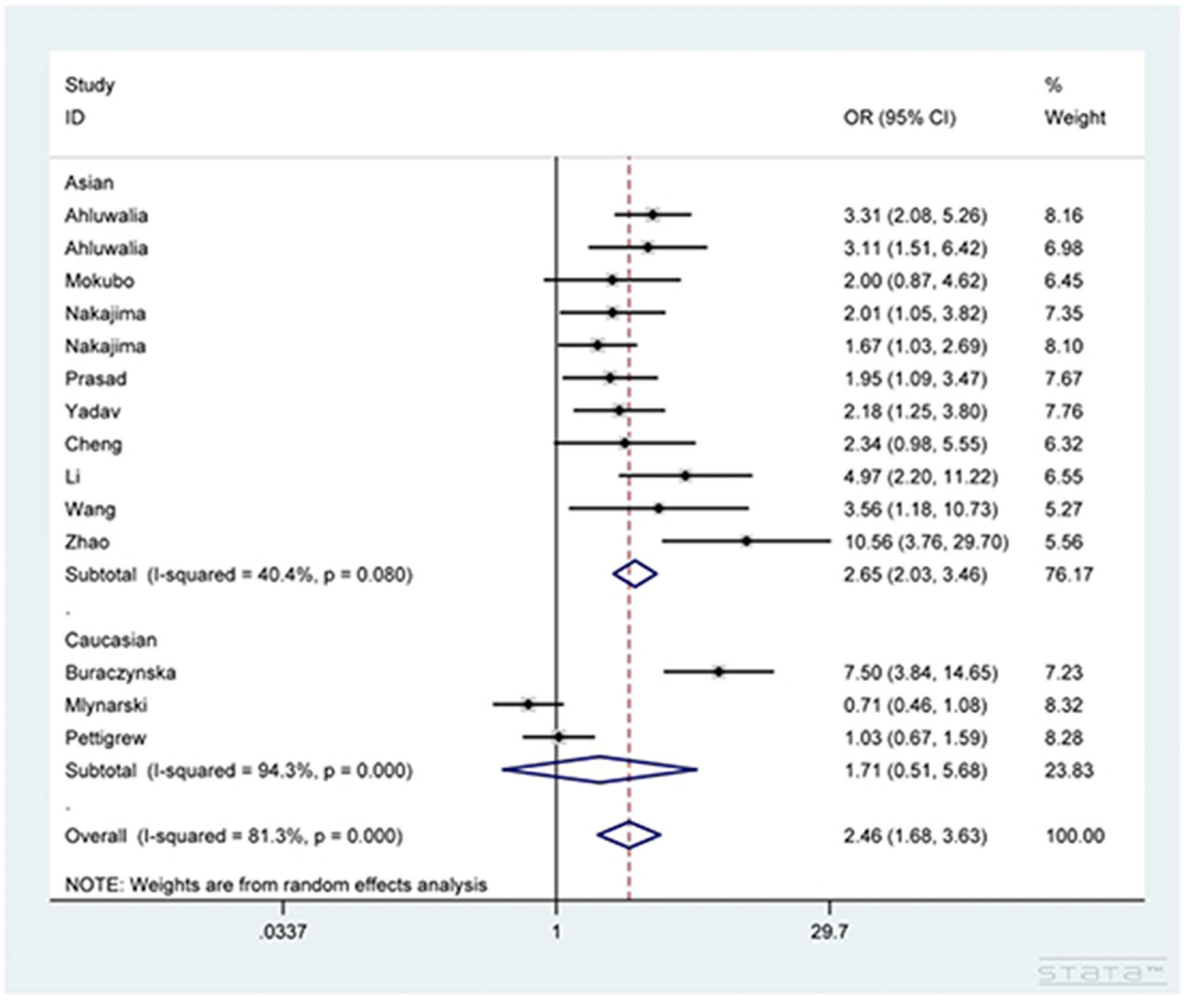
Forest plot for the association between CCR5 59029G/A polymorphism and diabetic nephropathy susceptibility under AA vs. GG contrast

**Figure 3:**
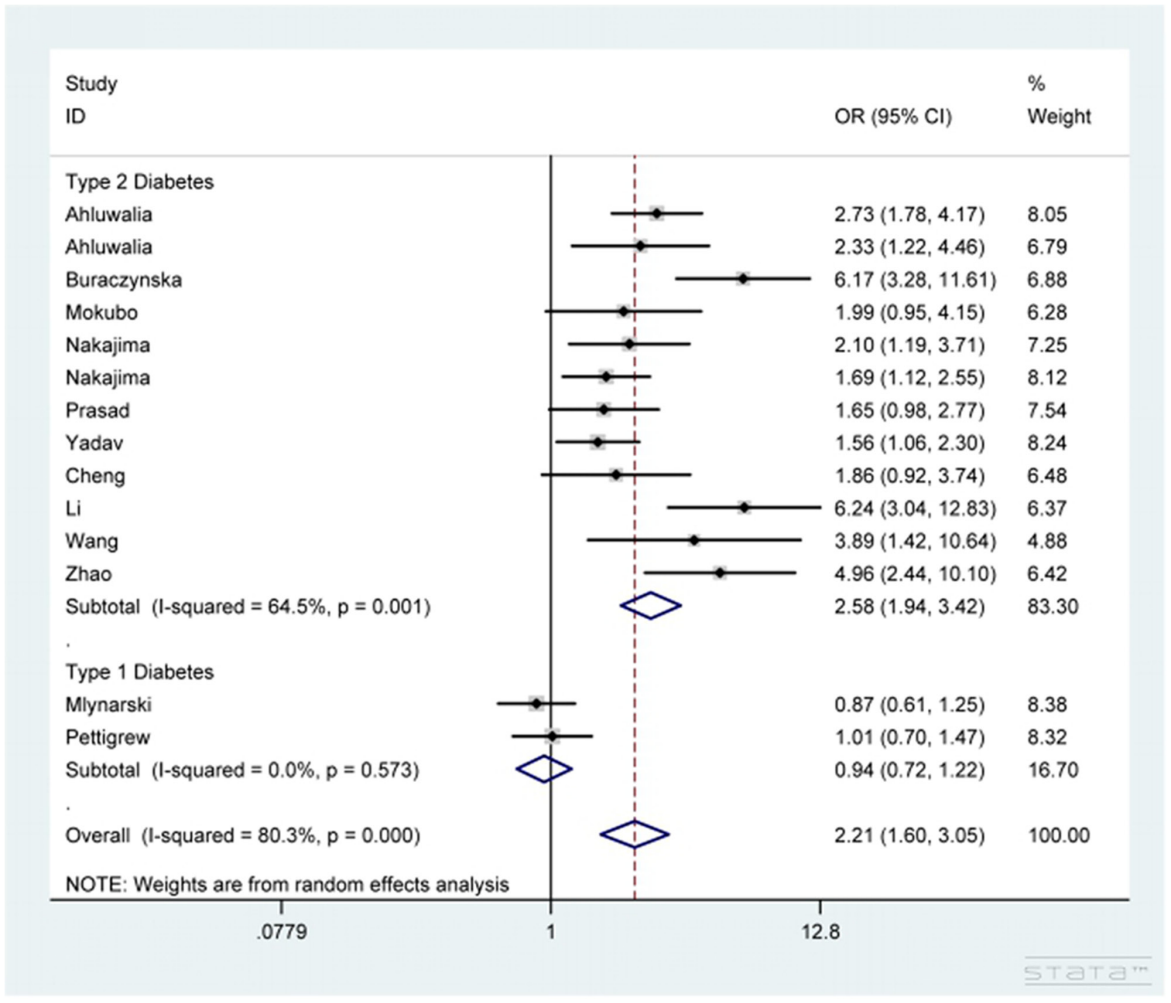
Forest plot for the association between CCR5 59029G/A polymorphism and diabetic nephropathy susceptibility under AA+GA vs. GG contrast after stratification analysis by type of diabetes mellitus

As shown in Table 2, *CCR5* 59029G/A polymorphism dramatically increased the susceptibility to DN in total analysis under the five genetic comparisons of AA vs. GG, AA+GA vs. GG, AA vs. GG+GA, A vs. G and GA vs. GG (OR=2.46, 95% CI=1.68-3.63 (Figure 2); OR=2.21, 95% CI=1.60-3.05 (Figure 3); OR=1.53, 95% CI=1.21-1.94; OR=1.63, 95% CI=1.32-2.01; OR=1.98, 95% CI=1.47-2.66). In addition, under the same five contrasts, a similar effect of the polymorphism was also observed in Asian (Figure 2) and type 2 diabetes mellitus (Figure 3) groups after subgroup analyses based on ethnicity and type of diabetes mellitus.

**Table 2:** *CCR5* 59029G/A polymorphism and diabetic nephropathy susceptibility

Group	No. of studies	Odds ratio (95% confidence interval) / *P* value for heterogeneity									
		AA versus GG		AA + GA versus GG		AA versus GG+GA		A versus G		GA versus GG	
Asian	11	2.65 (2.03, 3.46)	0.080	2.35 (1.82, 3.03)	0.019	1.70 (1.37, 2.12)	0.055	1.80 (1.47, 2.20)	0.000	2.04 (1.57, 2.64)	0.057
Caucasian	3	1.71 (0.51, 5.68)	0.000	1.69 (0.64, 4.47)	0.000	1.09 (0.66, 1.81)	0.001	1.16 (0.74, 1.79)	0.000	1.66 (0.69, 4.02)	0.000
Type 2 Diabetes	2	2.96 (2.19, 4.01)	0.007	2.58 (1.94, 3.42)	0.001	1.70 (1.40, 2.06)	0.077	1.79 (1.50, 2.14)	0.000	2.27 (1.70, 3.04)	0.005
Type 1 Diabetes	12	0.85 (0.59, 1.23)	0.222	0.94 (0.72, 1.22)	0.573	0.86 (0.61, 1.22)	0.135	0.92 (0.77, 1.11)	0.213	0.99 (0.75, 1.30)	0.908
Total	14	2.46 (1.68, 3.63)	0.000	2.21 (1.60, 3.05)	0.000	1.53 (1.21, 1.94)	0.000	1.63 (1.32, 2.01)	0.000	1.98 (1.47, 2.66)	0.000

### Heterogeneity test

In total analysis, significant heterogeneity was detected under all five genetic models, so the random-effects model was chosen for calculating pooled ORs. After stratified analyses by ethnicity and type of diabetes mellitus, the degree of heterogeneity significance was alleviated in some subgroups, indicating these two aspects might be related to the source of significant heterogeneity.

### Sensitivity analysis

**Figure 4:**
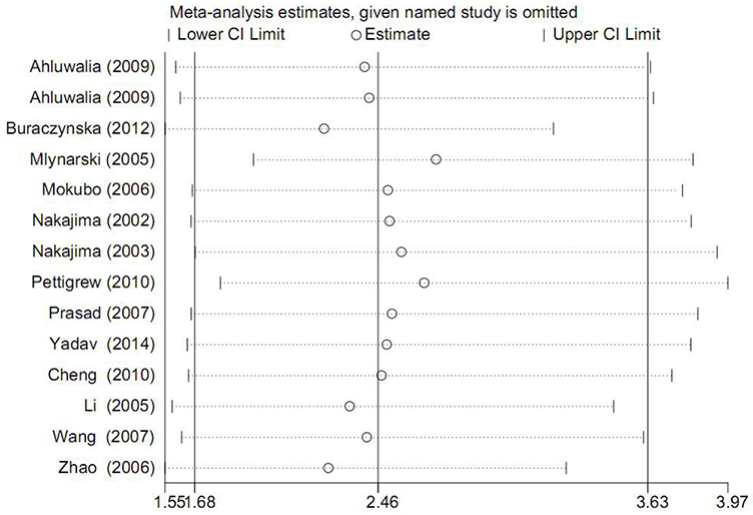
Forest plot for sensitivity analysis under AA vs. GG contrast

Sensitivity analysis was completed via deleting one single study each time and then re-calculating pooled ORs to observe alteration in final results. During this whole process, no material variation was detected after removing any one of the studies (Figure 4), showing the statistical robustness of our findings.

### Publication bias examination

**Figure 5:**
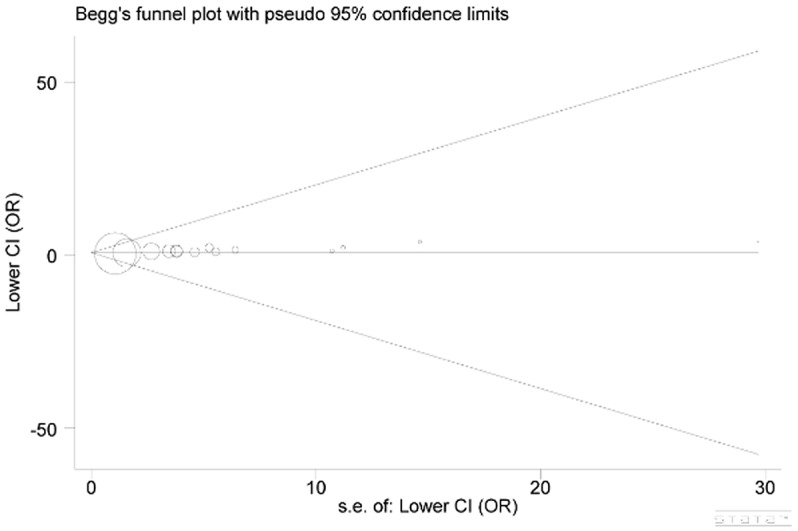
Begg’s funnel plot for publication bias

**Figure 6:**
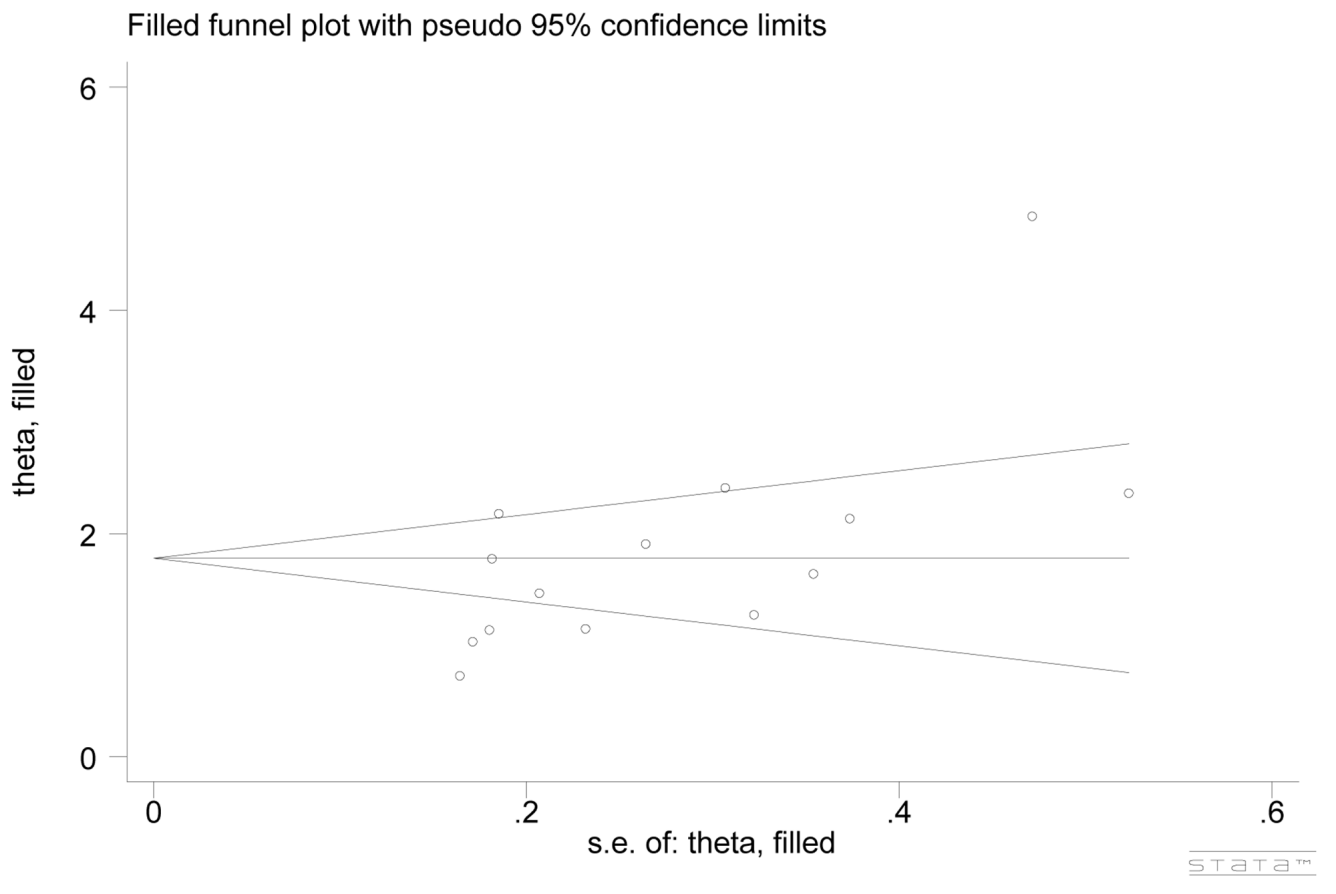
Begg’s funnel plot adjusted by non-parametric “trim and fill’ method

Publication bias across included studies was investigated with both Begg’s funnel plot and Egger’s test. As a result, the shape of funnel plots seemed asymmetric (Figure 5), implying the presence of significant publication bias. Furthermore, these results were statistically confirmed by evidence from Egger’s test (*P*=0.000). Such situation might be attributed to multiple aspects, such as studies with negative results more likely to be published in local rather than international journals, reports with null findings or small sample sizes having less chance to be published, as well as the interests of sponsors or beneficiaries. Non-parametric “trim and fill’ method was used to adjust publication bias and the results showed that no trimming was performed, the data was unchanged (Figure 6). The results demonstrated that the asymmetry of the funnel was not caused by the publication bias.

## DISCUSSION

As a serious complication of diabetes, DN is the leading cause of ESRD. Accompanied by high levels of pressure, sugar, lipids, protein and viscosity in blood as well as microcirculation disturbance, DN can result in the arteriosclerosis of kidney glomerulus, thus leading to edema and proteinuria. The incidence of DN is associated with many factors, such as high glucose, oxidative stress, metabolic disorder, and hemodynamics change. Besides, DN shows a familial aggregation tendency, indicating the important role of hereditary factors in its etiology. Nevertheless, the precise etiology of this disease still remains obscure at present. CCR5 is a G protein coupled receptor, and consists of 352 amino acids with a molecular weight of 40.6KD. It is expressed on the surface of mononuclear macrophages, dendritic cells and memory T cells, and is constituted by 7 transmembrane domains, extracellular N-terminal, extracellular and intracellular domains, and intracellular C-terminal. As a β chemokine factor, CCR5 plays vital roles in the chemotaxis, proliferation and immunoregulation of inflammatory cells. The polymorphism 59029G/A in promoter regions of *CCR5* gene can alter the activity of CCR5 protein, which has been reported to be correlated to the susceptibility of individuals to DN by various researchers.

For example, Ahluwalia et al. found a significantly higher frequency of the AA genotype of CCR 59029G/A polymorphism in patients with DN in both North and South Indians, showing the susceptible role of the genotype [22]. Additionally, in Caucasians of Polish origin, Buraczynska et al. found a risk-increasing effect of A allele on DN in patients with type 2 diabetic mellitus [23]. Besides, a similar influence of A allele and/or A+ genotype(s) of the polymorphism on susceptibility to DN was also replicated in several Chinese populations [31–34]. In contrast, Mlynarski et al. showed that in type 1 diabetic patients, male carriers of G allele had a 1.9-fold higher risk of developing DN compared with non-carriers [24]. Moreover, the study by Pettigrew et al. revealed no significant correlation of the polymorphism with the risk of DN either [28]. There were no conclusions on this issue.

To obtain a more reliable conclusion, we performed this meta-analysis based on previously published studies, and the results manifested a significant risk-enhancing effect of the polymorphism on the risk of DN in total analysis as well as Asian and type 2 diabetes mellitus groups under the five genetic contrasts of AA vs. GG, AA+GA vs. GG, AA vs. GG+GA, A vs. G and GA vs. GG.

There were some inevitable shortcomings in the present meta-analysis. First, the number of included studies was relatively small, which might affect the comprehensiveness of our findings. Meanwhile, the majority of included studies were based on Asian populations, which might introduce some selection bias. Second, the baseline characteristics like age and gender distribution, ethnicity, life styles, and family history in different studies might be statistically different, thus causing some clinical heterogeneity. Third, the accuracy of genotyping in the included studies could not be guaranteed. Among the 13 included studies, PCR-RFLP was the most frequently used method for genotyping. Compared with direct sequencing, PCR-RFLP might exhibit low accuracy. Furthermore, the detailed information about source of control and the diagnostic criteria of DN was not available in some included studies. Last but not least, the potential joint effects of the studied polymorphism with other relevant factors were not explored in this study. Additionally, the shape of funnel plots seemed asymmetric in the current study, but the analysis performed using non-parametric “trim and fill’ method indicated that publication bias was not responsible for the asymmetry. The model used for analysis might also heterogeneity to the final results [35, 36]. The multivariate model was needed to be constructed to estimate the genotype-disease associations. Further investigations will be required to address the above issues.

In conclusion, the results from the present meta-analysis provided additional evidences for the significant relationship of *CCR5* 59029G/A polymorphism with the susceptibility to DN. Considering the above-mentioned restrictions, our findings still need to be verified in future by studies with larger sample sizes and concerning potential gene-gene and gene-environment joint effects.

## MATERIALS AND METHODS

### Literature searching

The relevant publications were systemically searched from electronic databases of PubMed, EMBASE, BIOSIS and CNKI. Only the studies published in English or Chinese language were considered to be included. The combination of the following key terms were used for literature searching: “C-C chemokine receptor type 5” or “CCR5” or “CD195” or “CKR5”, “diabetic nephropathy” or “DN”, and “polymorphism” or “mutation” or “variant”. Meanwhile, other sources were also checked for the same purpose. Besides, the references of relevant articles were also screened for additional reports.

### Inclusion and exclusion criteria

All eligible articles must conform to the following criteria: (1) with a case-control design: the individuals in control group were Type 2 Diabetes or Type 1 Diabetes patients without nephropathy, while patients in case group were DN cases; (2) assessing the association between *CCR5* 59029G/A polymorphism and DN susceptibility; (3) offering sufficient information on genotype and/or allele frequencies in case and control groups; and (4) using validated genotyping method. Publications were eliminated from the present meta-analysis if any one of the following situations appeared: (1) adopting duplicated data on study participants; (2) animals as study objects; and (3) commentaries, case reports, review articles or conference abstracts. As for reports containing the same group of participants, we selected the one with the largest sample size.

### Data extraction

Data extraction was done independently by two investigators according to the same standard form. The major information included first author’s name, publication year, original country, ethnicity, type of diabetes mellitus, number of cases and controls, genotyping method, genotype and/or allele frequencies in case and control groups, and *P* value for Hardy-Weinberg equilibrium (HWE) in controls. If more than one study was incorporated in one single article, their data were abstracted separately.

### Quality analysis of included studies

In this meta-analysis, quality assessment of included studies was conducted using the criteria of Newcastle-Ottawa Scale (NOS). 0-4 scores means the poor quality and the article with 5-9 scores was the good quality study.

### Statistical analysis

STATA 12.0 software (Stata Corporation, College Station, TX, USA) was applied to complete all statistical analyses in this study. The strength of the relationship between *CCR5* 59029G/A polymorphism and DN susceptibility was evaluated through calculating pooled odds ratios (ORs) with their corresponding 95% confidence intervals (95% CIs). Between-study heterogeneity was detected with chi-square-based Q test, with *P*<0.05 as the significant level. When significant heterogeneity appeared (*P*_heterogeneity_<0.05), the random-effects model was employed to calculate pooled ORs, otherwise, fixed-effects model was used. Sensitivity analysis was performed through sequential omission of each included study to observe alteration in pooled ORs so as to test the stability of the final results. Begg’s funnel plot and Egger’s regression test were both adopted for publication bias examination among included studies. If publication bias was detected, Duval and Tweedie non-parametric “trim and fill’ method was used to adjust publication bias.
